# The Rapid Coronavirus Antibody Test: Can We Improve Accuracy?

**DOI:** 10.3389/fmed.2020.00569

**Published:** 2020-09-02

**Authors:** Ina P. Pavlova, Sujit S. Nair, Natasha Kyprianou, Ash K. Tewari

**Affiliations:** ^1^Department of Urology, Icahn School of Medicine at Mount Sinai, New York, NY, United States; ^2^The Tisch Cancer Institute, Icahn School of Medicine at Mount Sinai, New York, NY, United States; ^3^Department of Oncological Sciences, Icahn School of Medicine at Mount Sinai, New York, NY, United States; ^4^Department of Pathology, Icahn School of Medicine at Mount Sinai, New York, NY, United States

**Keywords:** SARS-CoV-2 antibodies, neutralizing antibodies, rapid tests, lateral flow assays, paper based analytical devices

## Introduction

We are at a critical stage in managing the response to the COVID-19 outbreak, which requires widespread access to fast and accurate testing. While PCR testing has been the backbone for COVID-19 diagnosis, now there is an urgent need for surveillance of at-risk asymptomatic populations. Antibody tests check for an antibody response to SARS-CoV-2 infection and are used to determine infection and case fatality rates, or potential immunity in recovered patients and in vaccine studies. Effective laboratory SARS-CoV-2 antibody technologies have been developed, and some were validated by the FDA to have Sensitivity (Se) and Specificity (Sp) as high as 99–100%^1^. For example, an IgG two-step ELISA test measures IgG responses to the recombinant receptor binding domain (RBD) of the SARS-CoV-2 spike protein (1). Positive samples are confirmed in a second step that measures IgG response to the whole spike protein (1), resulting in a 100% Sp (with 92.5% Se)^1^. However, while accurate, laboratory technologies are slow and rely on expensive equipment.

Rapid (minutes vs. hours) and instrument-free SARS-CoV-2 assays are commercially available, and some are already being used in surveillance studies. Debates about the recently reported infection rates in NYC (21.1% as of 04/23/20^2^), or in Santa Clara, CA [2.45% (2)], have raised questions regarding whether antibody testing is sufficiently accurate to guide medical or policy decisions. Recently, the COVID-19 Testing Project validated 10 rapid commercial tests in a head-to-head comparison with samples from 80 SARS-CoV-2 RT-PCR-positive, 108 pre-COVID-19 negative, and 52 recently negative patients (3). Many rapid tests performed worse than their manufacturer's specifications, raising questions about their quality and stability. Moreover, while high specificity is crucial for testing low prevalence population (estimated COVID-19 prevalence is only ~5%), only three out of 10 rapid tests had a Sp of >99%, while maintaining >90% Se (at >16 days after onset of symptoms) (3). More recently, the FDA started their own validation of 13 EUA approved antibody tests and found that only one of the validated rapid tests has a > 99% Sp (with a 95% Se)^1^. Introducing more stringent FDA criteria has driven the need for highly accurate rapid tests^3^. Here we summarize some of the limitations of rapid COVID-19 antibody tests and suggested ways for improvement.

## Isotype-Specific (IgM/IgG) Detection

After SARS-CoV-2 infection, IgM or IgG antibodies appear in the patient's blood that are specific for viral antigens to the spike glycoprotein such as the S1, S2 subunits, the receptor binding domain (RBD) or the nucleocapsid (N) protein (1). First, IgM becomes detectable within a few days and lasts several weeks after infection, followed by IgG detection. Currently, all rapid SARS-CoV-2 antibody tests rely on the ability of recombinant proteins of RBD, S1, S2, or the N domain of the SARS-CoV-2 spike protein to capture IgM or IgG antibodies in the patient's blood^3^ (4, 5). This isotype-specific detection (IgM or IgG) is time dependent; high sensitivity rates are achieved only at 3 weeks from symptom onset (3). For example, the COVID-19 Testing Project (3) showed that overall sensitivity of all validated rapid tests reached >80% Se only at >20 days of symptom onset (maintaining 95% Sp). None of the tests showed >80% Se at 6–10 days of symptom onset and only half showed >80% Se at 11–15 days of symptom onset.

Moreover, these validated rapid tests tend to have a higher Se for patients admitted to ICU compared to patients with milder disease (3). Recent clinical studies of antibody responses in patients with COVID-19 have associated higher IgG and IgM titers with worse disease outcome at all time points following the onset of symptoms (6), or with worse clinical readouts and older age (7). These findings suggest that rapid assay kits may favor the detection of higher IgG and IgM titers, and therefore perform better in more severe disease. In addition, while a growing number of studies report that SARs-CoV-2 antibodies are best detectable in infected people 3–4 weeks after symptom onset (8, 9), the antibody levels are lower and may have different kinetics in people with milder symptoms (10) and are is still largely unknown in asymptomatic people (9). This suggests that timing and choice of assays may have to be optimized depending on the populations to be tested. On the other hand, a study characterizing the neutralizing antibodies (Nabs) response in a cohort of COVID-19 recovered patients with mild symptoms, found a persistent Nabs response in 70% of recovered patients, with SARS-CoV-2-specific Nabs detected as early as 10–15 days after disease onset with kinetics aligned to that of binding antibodies (11). This suggests that Nabs detection could be performed in parallel to rapid isotype specific IgG and IgM detection to provide information about the functionality of the antibody repose and potential protection.

Rapid antibody tests capture binding IgG and IgM antibodies but not necessarily neutralizing antibodies (4, 5). Binding antibodies do not have the same neutralizing abilities or high affinity to the spike protein antigens as neutralizing antibodies (12). Recently, a SARS-CoV-2 surrogate virus neutralization test (sVNT) was developed that detects total neutralizing antibodies in an isotype-independent manner (13). This test utilizes the high-affinity interaction between the receptor binding domain (RBD) protein from the viral spike (S) protein and the host cell receptor ACE2 (hACE2) (14). Neutralizing antibodies inhibit this interaction by binding to the RBD protein prior to the virus-host interaction (12, 13). The sVNT test mimics this process by utilizing recombinant ACE2 and RBD proteins and detecting the % antibody-mediated inhibition (13). This test was validated to have 100% Sp (while maintaining 96% Se) in two patient cohorts. Moreover, its authors report superior sensitivity for low IgM/IgG titers compared to isotype-specific capture ELISA (13), suggesting that it can be used for testing in populations with lower levels of antibodies such as mildly symptomatic populations. However, currently its sensitivity is not validated by other studies and it is not yet adapted for rapid detection platforms.

## Lateral Flow Detection

Rapid SARS-CoV-2 antibody assays utilize lateral flow detection. Lateral flow tests are performed on a low-cost nitrocellulose strip which has assay reagents dried on the test zone. The target analyte diffuses from the sample deposition pad to the test zone by capillary action, and readout of the test zone is based on colorimetric detection (with gold nanoparticles conjugated to a detection antibody or recombinant protein), which eliminates the need for laboratory instruments. However, lateral flow tests are prone to variability due to many factors, including quality of the nitrocellulose and recombinant proteins, and their stability after drying. Moreover, simple lateral flow designs cannot perform multistep, sequential processes. Many laboratory assays rely on sequential washing and signal amplification steps for improved specificity and sensitivity. To enhance lateral flow designs, two-dimensional paper devices have been previously developed that allow for the timely delivery of multiple reagents to the test zone (15–17). These devices utilize capillary action and dried reagents, but their design incorporates additional compartments with detection, signal amplification or wash reagents so that fixed reagent volumes are delivered to the test zone in a sequential and controlled way. Such two-dimensional paper devices have previously been used successfully for the detection of antibodies against HPV and malaria (15–17), but not against SARS-CoV-2.

## New Testing Approaches

**Figure 1:**
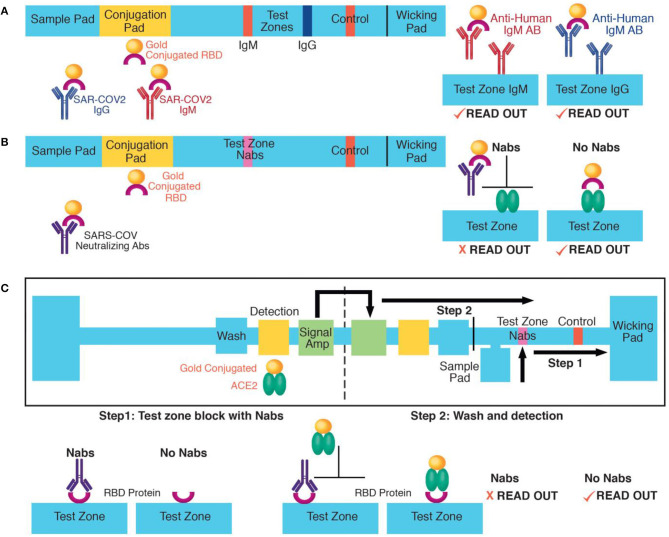
Approaches for rapid SARS-COV-2 antibody detection: **(A)** Example of IgM and IgG lateral flow detection. Antibodies move from the sample to the conjugation pad by capillary action where they bind dried recombinant RBD proteins conjugated to gold nanoparticles. Next, they are captured on the IgM or the IgG test zone. Aggregated nanoparticles at the test zones results in colorimetric readout. **(B)** A lateral flow detection of neutralizing antibodies (Nabs). A proposed application of the sVNT assay (13). Nabs move from the sample pad to the test zone by capillary action. At the conjugation pad, they bind to dried recombinant RBD proteins causing their neutralization. At the test zone, neutralized RBD proteins cannot bind to immobilized recombinant ACE2 and are washed out. In the absence of neutralizing antibodies, RBD proteins bind to ACE2 proteins at the test zone, causing colorimetric readout. **(C)** A multi-step, paper-based test for neutralizing antibodies. In step one, Nabs move from the sample pad to the test zone by capillary action and block recombinant RBD proteins immobilized at the test zone. **(C)** The left part of the device has a wash, detection (with dried recombinant ACE2 proteins conjugated to gold nanoparticles) and signal amplification pad. In step two, the device is folded to initiate to sequential delivery of a wash, detection, and signal amplification volume to the test zone. In the absence of neutralizing antibodies, the ACE2-gold nanoparticle complex binds to the test zone. The paper-based device schematic was adapted from (17).

One approach to improve the accuracy of rapid SARS-CoV-2 antibody tests is to adapt isotype independent assays, such as the sVNT test on lateral flow formats. Most current lateral flow tests have separate test zones for IgM and IgG detection, requiring two sets of capture and detection reagents (Figure 1A). However, a lateral flow sVNT assay would have only one test zone, simplifying reagent requirements (Figure 1B). We also suggest that a lateral flow sVNT test will have improved sensitivity, because it detects neutralizing antibodies with higher affinity to the recombinant RBD antigen than binding antibodies, optimizing capturing of the target analyte on the test strip. Further improvement could be achieved by integrating with a multi-step paper-based device (Figure 1C). This design allows for sequential delivery of a wash prior to the detection step (reducing false positives); and a final signal amplification step (optimizing sensitivity), while keeping a user-friendly, instrument-free, and disposable platform. In addition, testing a population with low prevalence of infection is challenging because even a highly specific assay can result in many false positive results. Therefore, an approach for decreasing false positives is to add confirmatory steps to lateral flow or paper-based devices, such as multiple test zones on the same test strip allowing binding to different viral epitopes (e.g., recombinant RBD test zone with confirmatory zones with the S1, S2, or N domains).

## Discussion

Results from SARS-CoV-2 testing influence the effective management of the current health crisis. Here we have outlined several factors that limit the accuracy of currently used rapid serological tests. First, most rapid tests utilize lateral flow detection with one-step delivery of the target analyte and detection reagents, which we argue limits their accuracy. Previously, multi-step paper-based platforms with time- and volume-controlled delivery of the target analyte and detection reagent have been validated for the detection of infectious diseases (15, 17). Exploiting such platforms for the detection of SARS-CoV-2 antibodies allows incorporating wash and signal amplification steps and sequential reagent delivery, currently lacking from rapid tests designs. We suggest that these additions will improve both sensitivity (due to signal amplification) as well as specificity (due to the wash between the sample and detection reagent delivery), while still maintaining a paper-based, disposable and cost effective platform. In addition, we argue that current rapid SARS-CoV-2 kits (based on isotype-specific IgM/IgG assays) favor detection of higher antibody titers. Specifically, since patients with more severe disease have higher titers (6, 7), we argue that these kits may have higher false negative rates when testing populations with mild disease as compared to those with severe symptoms and disease. Assays with better sensitivity for low titers such as such as the recently developed sVNT test for SARS-CoV-2 neutralizing antibodies (13) need to be applied on rapid detection platforms. Here we suggest that approaches for combining new antibody-based assays with multi-step, paper-based devices should be further exploited to improve the accuracy of current rapid SARS-CoV-2 testing. Formulation of these devices is straightforward and scalable; it requires only simple, low cost materials, such as nitrocellulose and glass fiber filters, and a laser cutter (17), as well as high quality recombinant SARS-COV-2 proteins, that are already commercially available (GenScript, Piscataway, NJ). Therefore, the proposed approaches will potentially provide a technology that is rapid and accurate, as well as scalable and low-cost, making it an attractive solution for mass screening of large populations.

Finally, SARS-CoV-2 antibody tests, even when highly accurate, would detect infection at best 2 to 3 weeks after symptom onset, which raises questions about how to optimize testing approaches for mildly or asymptomatic populations. For example, a study on the immune response of patients with mild disease report that IgG antibodies titers peaked around 24 days from symptom onset, suggesting that antibody testing should be done at least 3 to 4 weeks after symptom onset (11). This study also reports that in a cohort of people with suspected disease, only 36% of cases had a positive antibody test result. The authors suggest that this is partially due to insufficient time testing for mounting an antibody response, which emphasizes that improving detection of SARS-CoV-2 infection requires expanded viral load as well as antibody response testing. In line with these findings, we suggest that an optimized surveillance approach for mildly or asymptomatic populations could involve rapid testing for antibodies, as well as viral load. While PCR testing for viral load requires expensive laboratory equipment, many rapid and isothermal nucleic acid amplification approaches have been already developed for point of care applications. Moreover, recently the FDA approved the first SARS-CoV-2 antigen test that detects virus particles without needing PCR^4^. Therefore, one way to optimize screening of mildly or asymptomatic populations is to develop one integrated rapid paper-based test to detect both SARS-CoV-2 antibody status and virus load.

## Author Contributions

IP contributed to the conception, research, and writing and editing of manuscript. SN, NK, and AT were involved in the research and writing and editing of the manuscript. All authors contributed to the article and approved the submitted version.

## Conflict of Interest

The authors declare that the research was conducted in the absence of any commercial or financial relationships that could be construed as a potential conflict of interest.

## Footnotes

^1^https://www.fda.gov/medical-devices/emergency-situations-medical-devices/eua-authorized-serology-test-performance

^2^https://publish.twitter.com/?query=https%3A%2F%2Ftwitter.com%2FNYGovCuomo%2Fstatus%2F1253353968278876171&widget=Tweet

^3^https://www.fda.gov/news-events/fda-voices/insight-fdas-revised-policy-antibody-tests-prioritizing-access-and-accuracy

^4^https://www.fda.gov/news-events/press-announcements/coronavirus-covid-19-update-fda-authorizes-first-antigen-test-help-rapid-detection-virus-causes

## References

[1] Amanat F Stadlbauer D Strohmeier S Nguyen THO Chromikova V McMahon M. A serological assay to detect SARS-CoV-2 seroconversion in humans. Nat Med. (2020) 26, 1033–36. 10.1038/s41591-020-0913-5 32398876 PMC8183627

[2] Bendavid E Mulaney B Sood N Shah S Ling E Bromley-Dulfano R COVID-19 Antibody seroprevalence in Santa Clara County, California. medRxiv. (2020). 10.1101/2020.04.14.20062463 PMC7928865 33615345

[3] Whitman JD Hiatt J Mowery CT Shy BR Yu R Yamamoto TN. Test performance evaluation of SARS-CoV-2 serological assays. medRxiv. (2020). 10.1101/2020.04.25.20074856 32511497 PMC7273265

[4] Tang YW Schmitz JE Persing DH Stratton CW. The laboratory diagnosis of COVID-19 infection: current issues and challenges. J Clin Microbiol. (2020) 58:e00512–20. 10.1128/JCM.00512-20 32245835 PMC7269383

[5] Petherick A. Developing antibody tests for SARS-CoV-2. Lancet. (2020) 395:1101–2. 10.1016/S0140-6736(20)30788-1 32247384 PMC7270070

[6] Tan W Lu Y Zhang J Wang J Dan Y Tan Z. Viral kinetics and antibody responses in patients with COVID-19. medRxiv. (2020). 10.1101/2020.03.24.2004238232634129

[7] Jiang H Li Y Zhang H Wang W Men D Yang X. Global profiling of SARS- CoV-2 specific IgG/IgM responses of convalescents using a proteome microarray. medRxiv. (2020). 10.1101/2020.03.20.20039495 32665645 PMC7360742

[8] Wajnberg A Mansour M Leven E Bouvier NM Patel G Firpo A Humoral immune response and prolonged PCR positivity in a cohort of 1343 SARS-CoV 2 patients in the New York City region. medRxiv. (2020). 10.1101/2020.04.30.20085613

[9] Koopmans M Haagmans B. Assessing the extent of SARS-CoV-2 circulation through serological studies. Nat Med. (2020) 26:1171–72. 10.1038/s41591-020-1018-x 32719488

[10] Long Q Tang X Shi Q Li Q Deng H Yuan J. Clinical and immunological assessment of asymptomatic SARS-CoV-2 infections. Nat Med. (2020) 26, 1200–04. 10.1038/s41591-020-0965-6 32555424

[11] Wu F Wang A Liu M Wang Q Chen J Xia S Neutralizing antibody responses to SARS- CoV-2 in a COVID-19 recovered patient cohort and their implications. medRxiv. (2020). 10.1101/2020.03.30.20047365

[12] Iwasaki A Yang Y. The potential danger of suboptimal antibody responses in COVID-19. Nat Rev Immunol. (2020) 20, 339–41. 10.1038/s41577-020-0321-6 32317716 PMC7187142

[13] Tan CW Chia WN Qin X Liu P Chen MI Tiu C. A SARS-CoV-2 surrogate virus neutralization test based on antibody-mediated blockage of ACE2–spike protein–protein interaction. Nat Biotechnol. (2020). 10.1038/s41587-020-0631-z. [Epub ahead of print]. 32704169

[14] Zhou P Yang XL Wang XG Hu B Zhang L Zhang W. A pneumonia outbreak associated with a new coronavirus of probable bat origin. Nature. (2020) 579:270–3. 10.1038/s41586-020-2012-7 32015507 PMC7095418

[15] Fridley GE Le H Yager P. Highly sensitive immunoassay based on controlled rehydration of patterned reagents in a 2-dimensional paper network. Anal Chem. (2014) 86:6447–53. 10.1021/ac500872j 24882058 PMC4082385

[16] Fu E Liang T Spicar-Mihalic P Houghtaling J Ramachandran S Yager P. Two-dimensional paper network format that enables simple multistep assays for use in low-resource settings in the context of malaria antigen detection. Anal Chem. (2012) 84:4574–9. 10.1021/ac300689s 22537313 PMC3366194

[17] Grant BD Smith CA Castle PE Scheurer ME Richards-Kortum R. A paper-based immunoassay to determine HPV vaccination status at the point-of-care. Vaccine. (2016) 34:5656–63. 10.1016/j.vaccine.2016.09.021 27667331 PMC5075515

